# RNAi-Mediated Suppression of *Laccase2* Impairs Cuticle Tanning and Molting in the Cotton Boll Weevil (*Anthonomus grandis*)

**DOI:** 10.3389/fphys.2020.591569

**Published:** 2020-11-16

**Authors:** Alexandre Augusto Pereira Firmino, Daniele Heloísa Pinheiro, Clidia Eduarda Moreira-Pinto, José Dijair Antonino, Leonardo Lima Pepino Macedo, Diogo Martins-de-Sa, Fabrício Barbosa Monteiro Arraes, Roberta Ramos Coelho, Fernando Campos de Assis Fonseca, Maria Cristina Mattar Silva, Janice de Almeida Engler, Marília Santos Silva, Isabela Tristan Lourenço-Tessutti, Walter Ribeiro Terra, Maria Fátima Grossi-de-Sa

**Affiliations:** ^1^Embrapa Genetic Resources and Biotechnology, Brasília, Brazil; ^2^Max Planck Institute of Molecular Plant Physiology, Potsdam-Golm, Germany; ^3^Department of Cell Biology, Federal University of Brasília (UnB), Brasília, Brazil; ^4^Departamento de Agronomia/Entomologia, Universidade Federal Rural de Pernambuco (UFRPE), Recife, Brazil; ^5^Department of Cellular and Molecular Biology, Federal University of Rio Grande do Sul (UFRGS), Porto Alegre, Brazil; ^6^National Institute of Science and Technology – INCT PlantStress Biotech – Embrapa, Brasília, Brazil; ^7^Département Santé des Plantes et Environnement, Institut National de la Recherche Agronomique and Institut Sophia Agrobiotech, Sophia Antipolis, France; ^8^Department of Chemistry, University of São Paulo, São Paulo, Brazil; ^9^Department of Biological Sciences, Catholic University o Brasília (UCB), Brasília, Brazil

**Keywords:** *Laccase2*, cuticle tanning, insect pest control, gene silencing, RNAi

## Abstract

The cotton boll weevil, *Anthonomus grandis*, is the most economically important pest of cotton in Brazil. Pest management programs focused on *A. grandis* are based mostly on the use of chemical insecticides, which may cause serious ecological impacts. Furthermore, *A. grandis* has developed resistance to some insecticides after their long-term use. Therefore, alternative control approaches that are more sustainable and have reduced environmental impacts are highly desirable to protect cotton crops from this destructive pest. RNA interference (RNAi) is a valuable reverse genetics tool for the investigation of gene function and has been explored for the development of strategies to control agricultural insect pests. This study aimed to evaluate the biological role of the *Laccase2* (*AgraLac2*) gene in *A. grandis* and its potential as an RNAi target for the control of this insect pest. We found that *AgraLac2* is expressed throughout the development of *A. grandis* with significantly higher expression in pupal and adult developmental stages. In addition, the immunolocalization of the AgraLac2 protein in third-instar larvae using specific antibodies revealed that AgraLac2 is distributed throughout the epithelial tissue, the cuticle and the tracheal system. We also verified that the knockdown of *AgraLac2* in *A. grandis* resulted in an altered cuticle tanning process, molting defects and arrested development. Remarkably, insects injected with ds*AgraLac2* exhibited defects in cuticle hardening and pigmentation. As a consequence, the development of ds*AgraLac2*-treated insects was compromised, and in cases of severe phenotypic defects, the insects subsequently died. On the contrary, insects subjected to control treatments did not show any visible phenotypic defects in cuticle formation and successfully molted to the pupal and adult stages. Taken together, our data indicate that *AgraLac2* is involved in the cuticle tanning process in *A. grandis* and may be a promising target for the development of RNAi-based technologies.

## Introduction

Laccases (*p*-diphenol:dioxygen oxidoreductase, EC 1.10.3.2) are enzymes of the multi-copper oxidase (MCO) family, which also includes ascorbate oxidases, bilirubin oxidases and metal oxidases (ferroxidases, cuprous oxidases, and manganese oxidases) (Sakurai and Kataoka, 2007). Laccase enzymes exhibit p-diphenol oxidase activity and are widely distributed in nature, being found in bacteria, fungi, plants and insects. Laccase enzymes act on a large number of substrates and are involved in distinct biological processes in different organisms, such as pigmentation, protection against UV light and metal oxidation in bacteria, degradation of lignocellulose by fungi, lignification of the plant cell wall and plant response to abiotic and biotic stresses, and cuticle tanning (sclerotization and pigmentation) in insects (Singh et al., 2011; Yang J. et al., 2017; Janusz et al., 2020).

Two main *Laccase* genes, *Laccase1* (*Lac1*/*MCO1*), and *Laccase2* (*Lac2*/*MCO2*), have been described in insects. *Lac1* is expressed in tissues such as salivary glands, midgut and Malpighian tubules and has been implicated in lignocellulose digestion, detoxification of secondary plant compounds, ascorbate and iron homeostasis, and immune defense in insects (Gorman et al., 2008; Coy et al., 2010; Lang et al., 2012; Liu et al., 2015; Peng et al., 2015; Yang C.-H. et al., 2017; Wang et al., 2018; Zhang et al., 2018), while *Lac2* is expressed primarily in the epidermis and has been associated with insect cuticular pigmentation and hardening as well as melanization immune response (Arakane et al., 2005; Elias-Neto et al., 2010; Futahashi et al., 2011; Ye et al., 2015; Du et al., 2017; Nishide et al., 2020). Further, *Lac2* has been related to mechanisms of insecticide cuticular resistance. Some studies suggested that the overexpression of *Lac2* could increase cuticle thickness and consequently decrease the insecticide penetration in the organism and confer resistance to insecticides (Pan et al., 2009; Julio et al., 2017).

Two protein isoforms encoded by alternative splicing forms of the *Lac2* gene (*Lac2A* and *Lac2B*), which differ in the C-terminal region, were identified in the coleopteran *Tribolium castaneum* and the dipterans *Anopheles gambiae* and *Anopheles sinensis* (Arakane et al., 2005; Gorman et al., 2008; Du et al., 2017). Although both protein isoforms play a role in the cuticle tanning, Lac2A isoform appears to be the main determinant of the tanning process in the insects (Arakane et al., 2005).

The insect cuticle consists of a complex structure formed by chitin fibers, cuticular proteins, lipids and pigments secreted by the epidermal cells (Moussian, 2010). During cuticle tanning, the protein-protein and protein-chitin cross-linking are mediated by the action of cuticular diphenoloxidases. In this process, the oxidation of N-acetyldopamine (NADA) and N-β-alanyldopamine (NBAD) to *o*-quinones, catalyzed by Lac2, is essential for cuticle pigmentation and sclerotization, which may occur before and after each insect molt (Andersen, 2010).

RNA interference (RNAi) is a highly conserved cellular process of sequence-specific gene silencing present in eukaryotes, which has been explored as a tool for functional genomics studies and a pest control strategy (Baum et al., 2007; Mao et al., 2007; Zhang et al., 2015; San Miguel and Scott, 2016). Gene function analysis through RNAi-mediated silencing has been used to determine the biological roles of the *Lac2* gene and several studies have demonstrated that this gene is essential for cuticular pigmentation and hardening in diverse insect species, including coleopterans (Elias-Neto et al., 2010; Futahashi et al., 2011; Prentice et al., 2015; Christiaens et al., 2016; Du et al., 2017). Furthermore, *Lac2* dysfunction can lead to arrested development, molting defects, and insect mortality (Arakane et al., 2005; Prentice et al., 2015; Du et al., 2017; Nishide et al., 2020). Therefore, the importance of *Lac2* during insect development makes this gene a potential target for RNAi-based insect pest control technologies.

The cotton boll weevil, *Anthonomus grandis* Boheman (Coleoptera: Curculionidae), is the main insect pest of cotton crops in countries of Central and South America, especially in Brazil. It uses cotton flower buds and fruit bolls as a food source and a site for the development of its immature forms, causing direct damage to cotton fiber production and quality (Showler, 2008). The endophytic habit of *A. grandis* makes its control by chemical insecticides extremely difficult. However, this management strategy, which is directed against adult insects, has been the most efficient control method (Rolim and Netto, 2019). In the Cerrado, the largest cotton-producing region in Brazil, the number of insecticide applications during the growing season can vary between 15 and 26 depending on the infestation level, resulting in increased production costs (Miranda et al., 2015; Monnerat et al., 2019). Despite the efficacy of the chemical insecticides, their indiscriminate use can cause adverse environmental effects and lead to the emergence of resistant populations (Oliveira-Marra et al., 2019; Rolim and Netto, 2019). The serious damage to cotton crops caused by *A. grandis* attack along with the harmful side effects of the insecticides toward non-target organisms and the environment has prompted the development of innovative and sustainable strategies that can be employed in the management of this insect pest.

To gain a deeper understanding of the biological role of *AgraLac2* in *A. grandis* and evaluate whether it may be a suitable target gene for RNAi-mediated control methods, we evaluated the expression of the *AgraLac2* gene across the developmental stages of *A. grandis* as well as the expression of the AgraLac2 protein in larval tissues. In addition, loss-of-function analysis of the *AgraLac2* gene by dsRNA-induced silencing was performed.

## Materials and Methods

### Insect Rearing

*A. grandis* was reared under controlled temperature (27 ± 2°C), relative humidity (70 ± 10%) and photoperiod (14 light:10 dark). Insects were fed daily with an artificial diet (Monnerat et al., 2000).

### Identification, cDNA Cloning and Sequence Analyses of *AgraLac2*

tBLASTn analyses were performed to search for *Lac2* orthologue sequences in the *A. grandis* transcriptome (Firmino et al., 2013) using Lac2 sequences from *T. castaneum* (AAX84202.2 and AAX84203.2) as queries. Two orthologous sequences corresponding to the *Lac2* gene were retrieved from the *A. grandis* transcriptome (A_grandis_454_c23509 and A_grandis_454_rep_c1717). Thereafter, BLASTx searches were performed against the NCBI non-redundant protein database using *A. grandis* contigs to confirm their identity. To obtain a clone of *AgraLac2*, a partial sequence of *AgraLac2* was amplified from larval *A. grandis* cDNA and the PCR product was cloned into the PCR^TM^2.1 vector using the TA Cloning^®^ Kit (Invitrogen, Carlsbad, CA, United States) following the manufacturer’s protocol, and then sequenced to check the identity of the insert. Sequence alignment of the cloned *AgraLac2* sequence with *AgraLac2* contigs was performed to obtain a consensus sequence of *AgraLac2* (Supplementary Figure 1).

### Bioinformatic Analyses

Phylogenetic analysis with different insect Lac2 protein sequences was performed to evaluate possible orthology relationships among the predicted AgraLac2 protein (Supplementary Data 3) and the other selected Lac2 proteins from four different insect orders (Coleoptera, Diptera, Hymenoptera, and Lepidoptera). DELTA-BLAST searches were performed in public databases (NCBI,^1^) using the AgraLac2 protein sequence as a bait. DELTA-BLAST constructs a Position-Specific Scoring Matrix (PSSM) using the results of a Conserved Domain Database (NCBI) and searches a sequence database. Only complete protein sequences belonging to four insect orders were selected. Additionally, the selected sequences showed similarity to AgraLac2 greater than 80%, alignment coverage greater than 85% and *e*-value lower than or equal to 10^–40^. These parameters allowed the selection of 127 different Lac2 protein sequences from four different insects orders (42 from dipterans, 19 from coleopterans, 45 from hymenopterans, and 21 from lepidopterans). After the selection, all the sequences with the chosen outgroup (*Nannophya pigmaea*; accession number BBC20924.1) were aligned using the software Mafft (Mafft – version 7^2^) (Katoh et al., 2019) by applying the iterative refinement method with global alignment (G-INS-i) guided by a preliminary phylogenetic tree (default mode). In the following step, phylogenetic reconstruction was performed by the Maximum Likelihood method using the Randomized Axelerated Maximum Likelihood software (RAxML – version 8.2.12) (Stamatakis, 2014) with the options # autoMRE (the software decided how many bootstrap replicates must be run) and −m PROTGAMMAAUTO (the best protein substitution model was auto-selected). The best phylogenetic tree was analyzed and annotated using the online tool Interactive Tree of Life (iTOL – version 4^3^) (Letunic and Bork, 2019).

The search for the characteristic domains in the selected Lac2 protein sequences was carried out with the software HMMER v3.3.1 (Wheeler and Eddy, 2013), which is used to search for homologous sequences on databases using probabilistic models called Hidden Markov Models (HMMs) (Terrapon et al., 2012). The version of the database selected for the construction of the HMM profile was Pfam 33.1^4^. Domain sequences that had similarity with HMM profile with *e*-value less than or equal to 10^–20^ were selected. The same selected domain protein sequences identified with HMM analysis were also identified with MEME v5.1.1^5^ (Bailey et al., 2009), which included Cu-oxidase (PF00394.22), Cu-oxidase 2 (PF07731.14), and Cu-oxidase 3 (PF07732.15). The figure of protein domain consensus was obtained with WebLogo^6^.

### AgraLac2 Immunolocalization

To analyze the localization of the AgraLac2 protein, immunohistochemistry assays were performed. The gut of third-instar *A. grandis* larvae was removed, and the carcass was fixed in 4% paraformaldehyde, washed with 50 mM PIPES (Piperazine-N,N′-bis (2-ethanesulfonic acid) buffer, pH 7.0, and gradually dehydrated in ethanol. Fixation was performed at 4°C, and dehydration was conducted on ice. After dehydration, the tissues were placed in 50% ethanol-methacrylate mixture and kept overnight at 4°C. The tissues were embedded in 100% BMM (butyl-methyl-methacrylate) containing 0.5% benzoin ethyl ether at 4°C, and then polymerized under exposure to UV light for 6 h. Sections of the BMM blocks containing the carcasses of third-instar *A. grandis* larvae were cut to a thickness of 5 μm with an ultra-microtome. The sections were placed on poly-L-lysine coated slides containing distilled water and dried on a hot plate at approximately 50°C overnight. The slides containing the sections were used for immunolocalization assay. The sections were incubated in acetone for 30 min to remove BMM, and the adhered tissues were rehydrated in decreasing concentrations of ethanol. The slides were washed in PIPES buffer and blocked with blocking buffer (2% bovine serum albumin in PIPES buffer) for 3 h at room temperature. Then, the sections were incubated with an anti-AgraLac2 protein antibody (1:50 in blocking buffer) or with 2% bovine serum albumin for 1 h at room temperature and kept at 4°C overnight. Thereafter, the sections were incubated for 2 h at 37°C and washed with PIPES buffer for 30 min. An Alexa Fluor 488-conjugated anti-rabbit IgG secondary antibody (Invitrogen, Carlsbad, CA, United States) (1:300 in blocking buffer) was added, followed by incubation for 1 h at room temperature and then for 1 h at 37°C. Before use, the antibodies were incubated at 37°C for 30 min and centrifuged for 5 min at 13,000 rpm. The sections were washed with PIPES buffer for 30 min, and nuclei were stained with 4′,6-diamidino-2-phenylindole-dihydrochloride (DAPI, 1 mg/mL in distilled water) for 5 min at room temperature. Finally, the slides were washed quickly in distilled water to remove the excess of DAPI and mounted in 90% glycerol for observation using a Zeiss Axioplan 2 microscope with appropriate filters. A polyclonal antibody for the AgraLac2 protein was generated by immunization of rabbits using a synthesized peptide (CIGRSPDTSVKKINL-NH2) as the antigen (Genscript, Piscataway, New Jersey, United States). The AgraLac2 antiserum was purified using lyophilized protein A.

The immunostaining assays with alkaline-phosphatase (AP) were performed under the same conditions of the immunofluorescence assays until the step of the secondary antibody. At this point, instead of an Alexa Fluor 488-conjugated secondary antibody, anti-rabbit IgG secondary antibody conjugated with AP (Invitrogen, Carlsbad, CA, United States) was used. The AP-conjugated anti-rabbit IgG secondary antibody (1:2000 in blocking buffer) was added, followed by incubation for 1 h at room temperature and then for 1 h at 37°C. Thereafter, the sections were washed with TPBS (tris phosphate buffered saline) buffer for 30 min, and the substrate for AP reaction (5-bromo-4-chloro-3-indolyl-phosphate/nitro blue tetrazolium: BCIP/NBT) was added and incubated at room temperature until color development. The reaction was stopped by incubation with PBS washing buffer for 20 min. The sections were imaged with a digital camera (Axiocam, Carl Zeiss, Jena, German). Pre-immune serum was used as a negative control.

Additionally, morphological analysis of larval tissues was performed to aid in cuticle and epidermis recognition and avoid data misinterpretation. The tissues were fixed and dehydrated as described previously. Then, the tissues were embedded in Technovit 7100 (Heraeus Kulzer, Wehrheim, Germany) as described by the manufacturer. The embedded tissues were sectioned (3 μm), stained in 0.05% toluidine blue, and imaged with a digital camera (Axiocam, Carl Zeiss, Jena, Germany).

### dsRNA Synthesis

Gene-specific primers designed using BLOCK-iT^TM^ RNAi Designer software^7^ containing the T7 promoter at 5′ end were used to amplify the DNA template for dsRNA synthesis (Table 1). The ds*AgraLac2* was designed to target a fragment of 332 bp length, starting at nucleotide position 785 and ending at nucleotide position 1114 of the cloned *AgraLac2* transcript sequence (Supplementary Figure 1). DNA template was PCR amplified from *A. grandis* adults and larval cDNA using the gene-specific primers. The PCR product was cloned into the pGEMT-easy vector (Promega, Madison, Wisconsin, United States) and sequenced to verify the identity of the amplified fragment. After the confirmation of specific target amplification, the dsRNA was synthesized from 500 ng of the PCR product using MEGAscript^®^ T7 High Yield Kit (Ambion, Austin, TX, United States) according to the manufacturer’s instructions and purified by phenol-chloroform extraction followed by isopropanol precipitation. dsRNA targeting the *green fluorescent protein* gene (dsGFP, 240 bp) was purchased from agroRNA (Seoul, South Korea) and purified by phenol-chloroform extraction.

**TABLE 1:** Primers used for dsRNA synthesis and RT-qPCR analyses.

Primer identification	Sequence 5′-3′	Amplicon size (bp)
dsRNA_Ag_Lac2_Fwd	**TAATACGACTCACTATAGGGG**	332
	CTCCGCTTCTATCTCAGT	
dsRNA_Ag_Lac2_Rv	**TAATACGACTCACTATAGGGG**	
	CAATGGTGTCTTTACCG	
qPCR_Ag_Lac2_Fwd	GGTTGATGAAGTTCAACA	192
qPCR_Ag_Lac2_Rv	GCAATGGTGTCTTTACCG	
qPCR_Ag_β-tubulin_Fw	GGTTGCGACTGTTTACAAGG	156
qPCR_Ag_β-tubulin_Rv	GCACCACCGAGTAAGTGTTC	
qPCR_Ag_gapdh_Fwd	AGATCGTCGAGGGTCTGATG	166
qPCR_Ag_gapdh_Rv	AAGGCGGGAATGACTTTACC	

*Bold letters represent the T7 promoter sequence.*

### RNAi Experiments

To evaluate the effects of *AgraLac2* gene knockdown on *A. grandis* development and mortality, we performed RNAi bioassays by microinjection of ds*AgraLac2*. Third-instar larvae were anesthetized on ice and injected into the dorsal region with 1 μL (500 ng) of ds*AgraLac2* using a 10 μL Hamilton syringe (Hamilton Co., Reno, United States). Water (mock) and ds*GFP* were used as negative controls. After injection, the larvae were maintained at 26 ± 2°C, relative humidity 60 ± 10, photoperiod (12 light:12 dark) and fed on an artificial diet. The mortality rates and morphological phenotypes of the insects were monitored for 30 days. The experiment was repeated three times as independent biological replicates, each consisting of 20 larvae. For RT-qPCR assays, 20 larvae were injected with ds*AgraLac2*, ds*GFP* or water. Two to three biological samples per treatment were collected for transcript analysis of *AgraLac2*. Larvae were collected at day 2 and 20, while pupae/adults were collected at day 14. Each sample consisted of a pool of three to five larvae or three pupae/adults.

### RNA Extraction, cDNA Synthesis and RT-qPCR Analysis

Total RNA was extracted from *A. grandis* insects using TRIzol reagent (Invitrogen, Carlsbad, CA, United States) according to the manufacturer’s instructions. The RNA samples were treated with 2 U of DNase I RNase-free (Ambion, Austin, TX, United States) for 30 min at 37°C to remove residual DNA contamination. The concentration of RNA samples was evaluated with a Qubit^®^ 2.0 Fluorometer using the Quant-iT RNA Assay Kit (Invitrogen, Carlsbad, CA, United States). RNA integrity and purity were also assessed by 1.5% agarose gel electrophoresis. Subsequently, total RNA (500 ng) was reverse transcribed to cDNA using the Superscript III^TM^ First-Strand Synthesis SuperMix Kit (Invitrogen, Carlsbad, CA, United States) according to the manufacturer’s instructions. The RT-qPCR reaction mix included 2.5 μL of SYBR Green Rox Plus PCR Mix (LGC Biotecnologia, Cotia, SP, Brazil), 2 μL of cDNA diluted 40×, 4.7 μL of double-distilled water and 0.4 μL of each primer (0.2 μM), in a total volume of 10 μL. RT-qPCR reactions were performed on a 7500 Fast Real-Time PCR System (Applied Biosystems, Foster City, CA, United States) under the following cycling conditions: 95°C for 10 min, followed by 40 cycles at 95°C for 15 s and 60°C for 1 min. At the end of each run, a melting curve analysis ranging from 60 to 94°C (0.5°C/1 s) was performed to ensure the specificity of amplification. A non-template control (NTC) and non-reverse transcriptase (NRT) using water and RNA as templates, respectively, were included in each run for each gene. The RT-qPCR assays were performed using two to three biological replicates and three technical replicates. The Ct values and efficiency of each primer pair were estimated using the Real-time PCR Miner software^8^ (Zhao and Fernald, 2005). The relative expression level of the target gene was calculated with qBasePlus software (Biogazelle, Zwijnaarde, Belgium) using the E^–ΔΔCt^ method (Pfaffl, 2001). *Glyceraldehyde 3-phosphate dehydrogenase* (*Gapdh*) and *β-tubulin* (*β-tub*) were used as reference genes for normalization. For *AgraLac2* gene expression analysis in different developmental stages of *A. grandis*, three biological replicates of eggs, larvae (1st – 3rd instar), pupae and 10 days old adults (females or males) were used.

### Statistical Analyses

All statistical analyses were performed with Sigma Plot version 12.0. Student’s *t*-test was used to compare two groups. One-way analysis of variance (ANOVA) followed by Tukey’s HSD test was used to compare more than two groups. A *P*-value < 0.05 was considered to be statistically significant.

## Results

### Identification, Cloning and Sequence Analyses of *AgraLac2*

We identified two contigs in the *A. grandis* transcriptome (contig A_grandis_454_rep_c1717 and contig A_grandis_454_rep_c23509) (Supplementary Data 1) putatively encoding Lac2. Significant hits to *A. grandis* contigs were retrieved using BLASTx against the NCBI non-redundant protein database (Supplementary Table 1). Based on the *AgraLac2* contigs, specific primers were designed, and a partial cDNA sequence of *AgraLac2* was cloned. The consensus sequence of the cloned fragment and *AgraLac2* contigs was identified as *Lac2* gene by BLASTx, confirming the identity of the sequence. The partial cDNA *AgraLac2* sequence has been deposited in the NCBI under the accession number MW029454.

### AgraLac2 Phylogenetic Analysis

**FIGURE 1:**
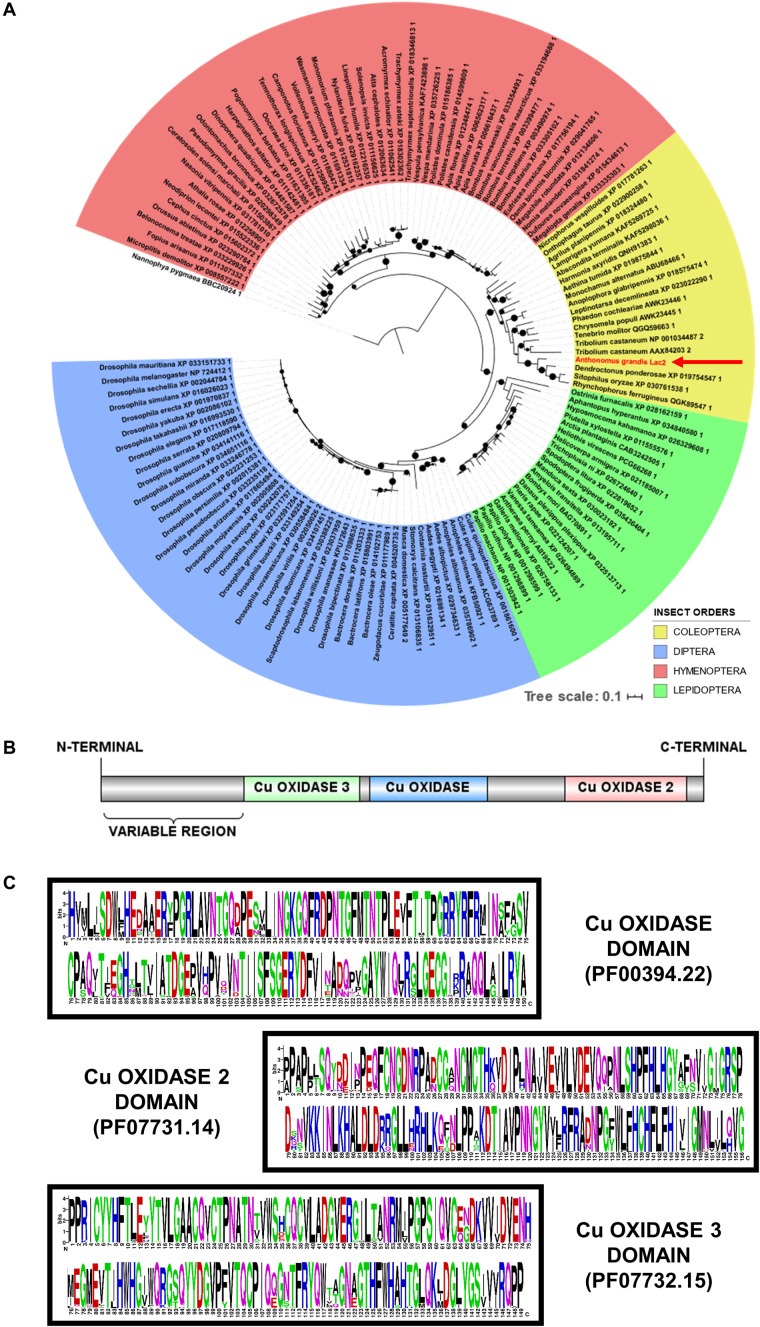
Laccase 2 protein in four different insect orders. In order to characterize *in silico* the protein sequence of *A. grandis* Laccase2 (AgraLac2), phylogenetic analyses **(A)** and domain analyses **(B,C)** were performed with 127 different Lac2 protein sequences from four different insect orders (42 from dipterans, 19 from coleopterans, 45 from hymenopterans and 21 from lepidopterans). **(A)** Maximum likelihood analysis of the AgraLac2 protein with its putative orthologues. The AgraLac2 sequence is highlighted in red (red arrow). The black circles present in the clades of the phylogenetic tree represent the Bootstrap values. The black circles with the smallest diameter represent the bootstrap value equal to 70, while the black circles with the largest diameter represent the bootstrap value equal to 100. Bootstrap values between this range are represented by black circles with increasing diameter. *Nannophya pigmaea* (BBC20924.1) is the outgroup. **(B)** Schematic representation of Lac2 protein from the analyzed insects (including AgraLac2), where an N-terminal region with variable sequence (absent in AgraLac2, since this study identified only partial AgraLac2 sequence) and three multicooper oxidase domains are highlighted: Cu-oxidase (PF00394.22; blue box), Cu-oxidase 2 (PF07731.14; pink box), and Cu-oxidase 3 (PF07732.15; green box). **(C)** Consensus sequence of three multicooper oxidase domains characteristic of insect Lac2 proteins. The consensus sequence was obtained from 127 different Lac2 proteins selected for this study.

An important step in the RNAi studies is the *in silico* characterization of the target sequence. Even with the advances achieved with traditional homology tools, phylogenetic analyses are still the most indicated and the most precise for predicting orthology relationships among sequences, since the quality of the functional annotation of genes deposited in public databases is not always accurate. For this purpose, phylogenetic trees are constructed with sequences whose function has been previously validated. According to the literature, Lac2 has been well characterized in *Manduca sexta* and *T. castaneum* (Arakane et al., 2005; Dittmer et al., 2009). Thus, as shown in Figure 1A, the clustering of the AgraLac2 protein sequence in the same group as the sequences of *M. sexta* and *T. castaneum* is a strong indication of orthology among these proteins that possibly share the same function. Furthermore, the phylogenetic analysis of Lac2 proteins from species of four orders of holometabolous insects (Coleoptera, Diptera, Hymenoptera, and Lepidoptera) showed that these sequences underwent a small evolutionary pressure since these protein sequences probably underwent few non-synonyms mutations during the evolution. This hypothesis can also be confirmed with the identification and *in silico* evaluation of the main protein domains of these sequences. According to the analyses of hidden Markov models (HMMs) with the Pfam database, the Lac2 from the insects analyzed are composed of 3 domains multicooper oxidases (Cu-oxidase 3 – PF07732.15, Cu-oxidase – PF00394.22, and Cu-oxidase 2 – PF07731.14), which have a structural conformation similar to cupredoxin: a beta-sandwich with 7 strands in 2 sheets, arranged in Greek-key beta-barrel (Messerschmidt and Huber, 1990; Ouzounis and Sander, 1991; Roberts et al., 2002). In addition to these domains, an N-terminal region with variable sequence composed by amino acids that possibly are associated with membranes was identified (Figure 1B). The multicooper domains identified in the analyzed sequences showed low variability, as can be seen in the consensus sequences in the Figure 1C. This small variability is a strong indication that Lac2 has great importance in the development of the analyzed insect species, including *A. grandis*, and that large variations in its sequence/structure may be deleterious.

### *AgraLac2* Expression Throughout the Development of *A. grandis* and Localization of AgraLac2 Protein

**FIGURE 2:**
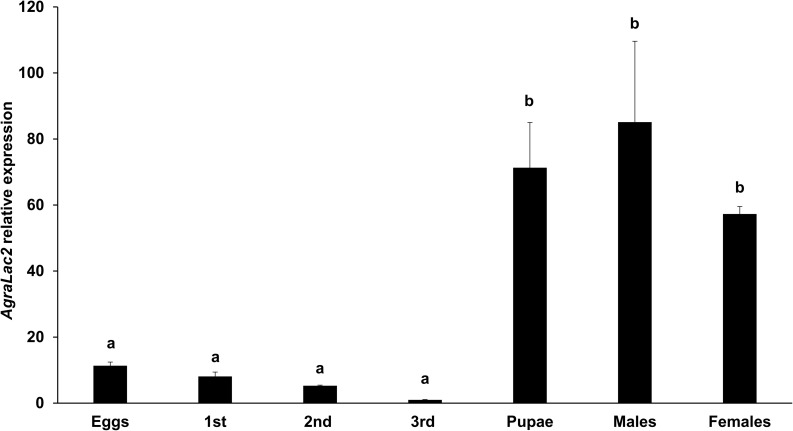
Expression profile of *AgraLac2* across the developmental stages of *A. grandis*. Relative transcript levels of the *AgraLac2* gene in eggs, larvae (1st – 3rd instar), pupae, male adults and female adults evaluated by RT-qPCR. The data were normalized using *gapdh* and *β-tub* as reference genes. Values shown are the means and standard errors (±SE) of three biological replicates each with three technical replicates. Data were analyzed by one-way ANOVA followed by a post hoc multiple comparisons test (Tukey’s HSD test). Treatment groups with different letters are significantly different (*P* < 0.05).

**FIGURE 3:**
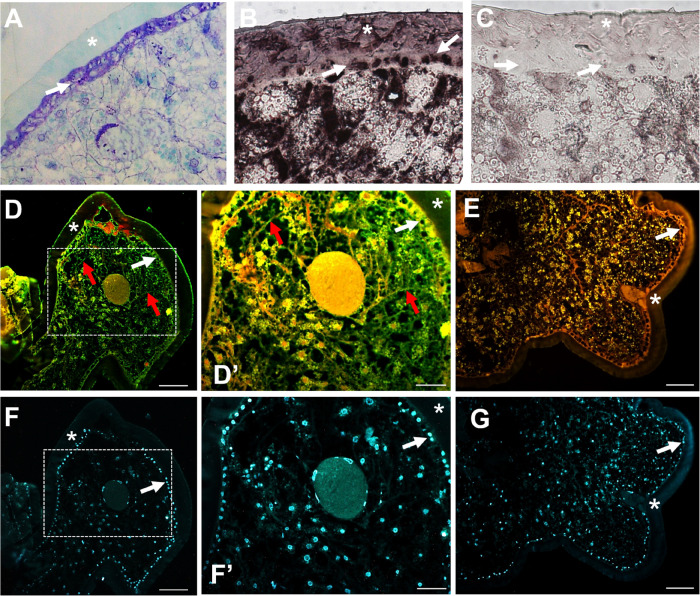
Localization of the AgraLac2 protein in third-instar larvae of *A. grandis*. **(A)** Section of larval tissues stained in 0.05% toluidine showing the cuticle and epidermis. **(B)** Immunostaining showing the localization of AgraLac2 protein. For immunostaining assays, the third-instar larvae were fixed and embedded in methacrylate resin. Thin sections were probed with primary rabbit anti-AgraLac2 polyclonal antibody and AP-conjugated anti-rabbit IgG secondary antibody and detected colorimetrically. **(C)** Negative control for immunostaining was performed through incubation with the pre-immune serum of rabbit immunized with AgraLac2 peptides. Scale bar = 10 μm. **(D,D’)** Immunolocalization of the AgraLac2 protein. For fluorescent immunolocalization assays, sections of larval tissues were incubated with primary rabbit anti-AgraLac2 polyclonal antibody. The anti-AgraLac2 antibody was detected with an Alexa Fluor 488-conjugated anti-rabbit IgG secondary antibody (green). **(E)** Negative control for immunolocalization, which was performed through incubation with PIPES buffer and BSA. **(F,F’,G)** Nuclei were stained with DAPI (blue). **(D’,F’)** Amplified images of white boxes shown in D and F. Scale bar = 100 μm. Asterisks indicate the larval cuticle. White arrows indicate the monolayer of epidermal cells below the larval cuticle. Red arrows indicate the tracheal system.

The transcriptional analysis of *AgraLac2* demonstrated that its expression was variable across the developmental stages of *A. grandis*. *AgraLac2* was highly expressed in pupae and adults (males and females), whereas the lowest expression levels were observed in larvae and eggs (Figure 2). In the sections stained with toluidine blue, we observed a typical cuticle and the underlying epidermal cells where the Lac2 is mainly synthesized (Figure 3A). To determine the localization of the AgraLac2 protein in the third-instar larvae of *A. grandis*, immunostaining and immunohistochemical assays were conducted. Our results indicated that AgraLac2 protein was localized mainly in the cuticle, epidermal cells and tracheal system, as demonstrated by the AP staining reaction (Figure 3B) or green fluorescence (Figures 3D,D’), while the control sections did not yield any staining signals (Figure 3C) or fluorescence signals (Figure 3E). Nuclei were stained with DAPI and could be visualized by blue fluorescence (Figures 3F,F’,G). We observed in the Figure 3B that besides the cuticle and epidermal cells, other regions of the section were stained. However, it was not possible to accurately determine through the immunostaining analyses what cells or structures correspond to these other stained regions. We speculate that it is the tracheal system as could be seen in the immunolocalization analyses (Figures 3D,D’).

### Functional Analysis of the *AgraLac2* Gene by RNAi

**FIGURE 4:**
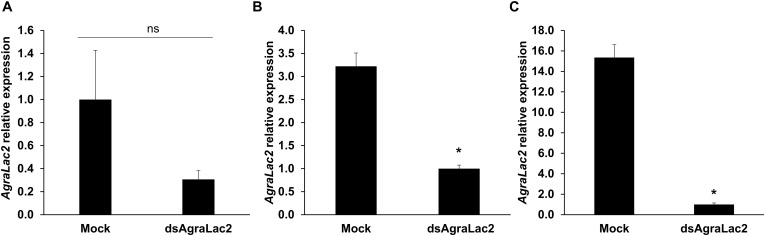
Relative transcript levels of *AgraLac2* after dsRNA exposure. Relative transcript levels of *AgraLac2* in *A. grandis* larvae at day 2 **(A)** and at day 20 **(B)**, and in *A. grandis* pupae/adults at day 14 **(C)** after water (mock) or ds*AgraLac2* injection were evaluated by RT-qPCR. The expression data were normalized using *gapdh* and *β-tub* as reference genes. Values shown are the means and standard errors (±SE) of two-three biological replicates each with three technical replicates. Data were analyzed by Student’s *t*-test. Significant differences are indicated with asterisks (*P* < 0.05); ns, not significant.

**FIGURE 5:**
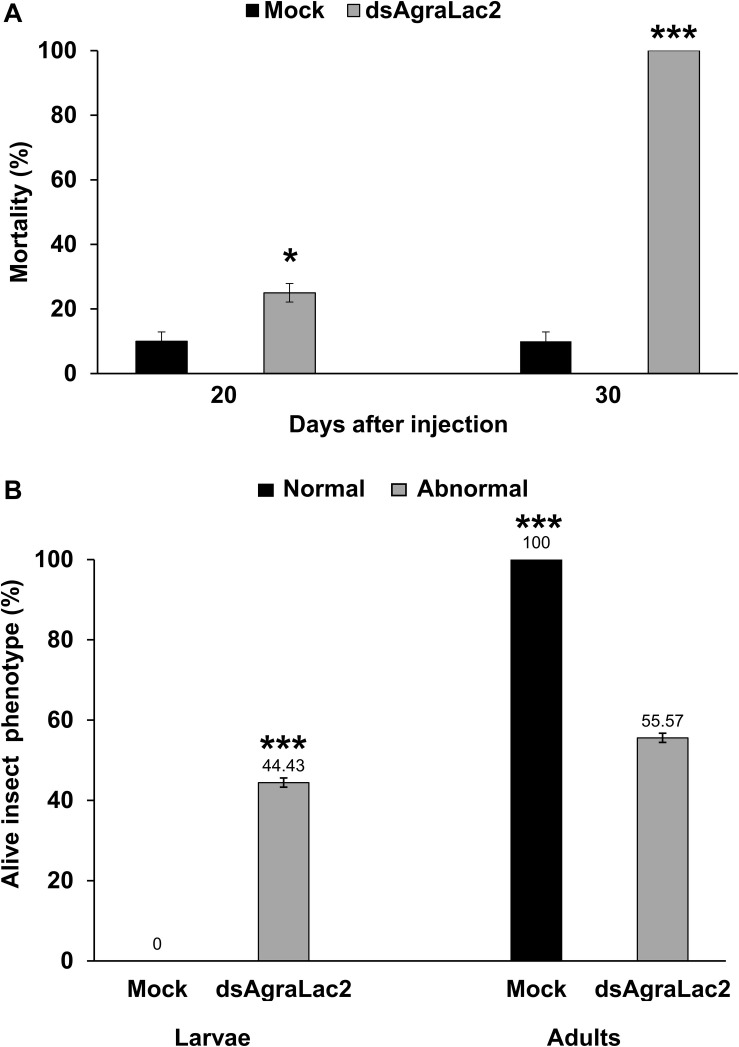
Effect of *AgraLac2* knockdown on *A. grandis* mortality and morphological phenotypes. Percent mortality at days 20 and 30 **(A)** and normal/abnormal phenotypes at day 20 **(B)** of the insects treated with *dsAgraLac2* or mock. Values shown are the means and standard errors (±SE) of three biological replicates. Data were analyzed by Student’s *t*-test. Significant differences are indicated with asterisks (*P < 0.05; ***P < 0.001).

To examine the function of *AgraLac2* in *A. grandis*, we performed dsRNA-mediated silencing of the *AgraLac2* transcripts. Two days after ds*AgraLac2* injection, we observed a trend toward decreased *AgraLac2* expression in *A. grandis* larvae compared to the controls, although the differences were not statistically significant (Figure 4A and Supplementary Figure 2). When the expression of *AgraLac2* was evaluated in pupae/adults and larvae with abnormal phenotypes 14 and 20 days after ds*AgraLac2* injection, respectively, we observed that the transcript levels were significantly reduced by 93.5 and 68.9%, indicating a strong and persistent suppression of the *AgraLac2* expression (Figures 4B,C). The knockdown of *AgraLac2* led to a slight but significant increase in the mortality rate of the insects compared to the control at day 20. However, the mortality of the insects microinjected with ds*AgraLac2* reached 100% at day 30, while the mortality rate of the control insects remained 10% as observed at day 20 (Figure 5A). We also observed that *AgraLac2* silencing seriously affected the insect morphology and cuticle tanning, causing incomplete molting from the pupa into the adult stage and arrested development. Among the insects that survived until day 20, we observed that 100% of the larvae from the control treatment reached the adult stage and exhibited a normal phenotype, whereas 44.4% of the larvae injected with ds*AgraLac2* remained at the larval stage, and 55.6% emerged into the adult stage, but both larvae and adults displayed an abnormal phenotype (Figure 5B).

**FIGURE 6:**
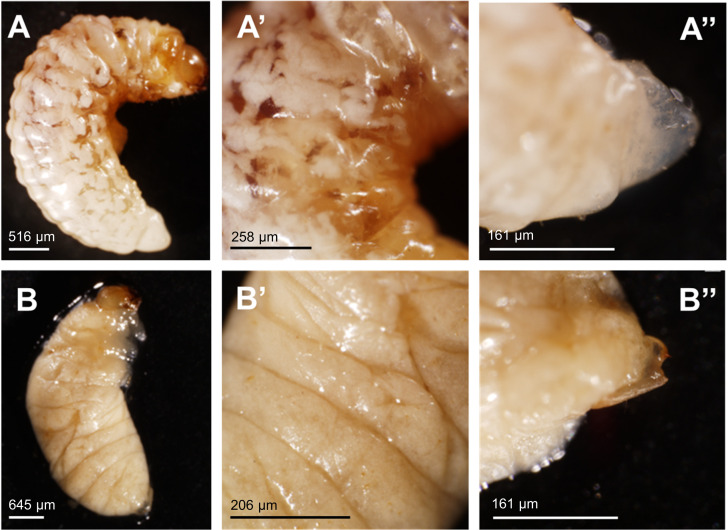
Morphological phenotype of *A. grandis* larvae induced by the injection of dsRNA targeting the *AgraLac2* gene. Third-instar larvae of *A. grandis* were injected with 500 ng of ds*AgraLac2* or water (mock), and the phenotypic effects were observed at 20 days after injection. A normal phenotype was observed in larvae from the control treatment **(A,A’,A”)** and an abnormal phenotype was observed in larvae treated with ds*AgraLac2* **(B,B’,B”)**. Representative images of insects were captured with a SZ61TR stereomicroscope (Olympus).

**FIGURE 7:**
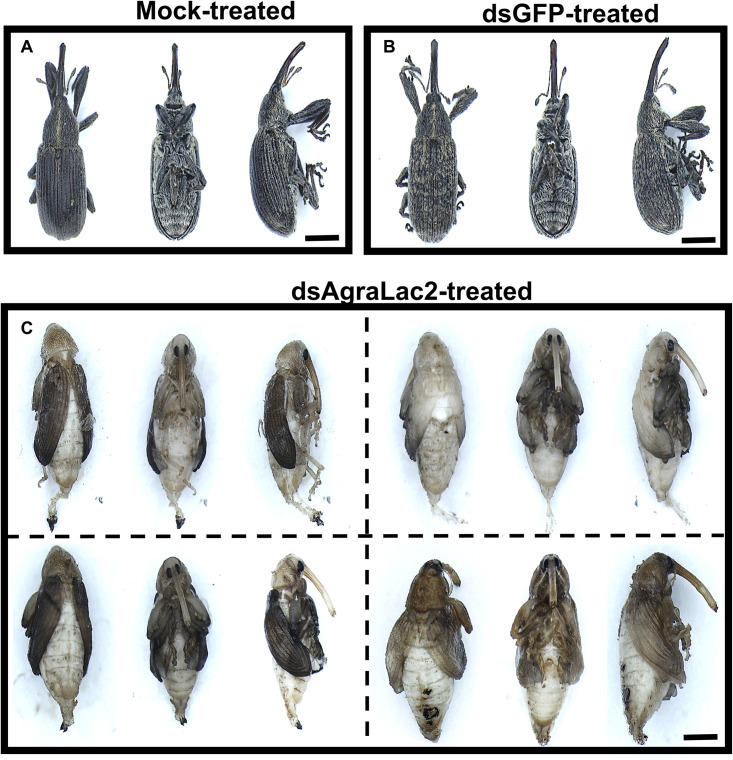
Morphological phenotype of *A. grandis* adults induced by the injection of dsRNA targeting the *AgraLac2* gene. Third-instar larvae of *A. grandis* were injected with 500 ng of ds*AgraLac2*, ds*GFP* or water (mock), and the phenotypic effects were observed at 20 days after injection. Dorsal, ventral and latero-ventral views of *A. grandis* subjected to the mock **(A)**, ds*GFP* **(B)** or ds*AgraLac2* **(C)** treatment at 20 days after injection. Insects from the control treatments **(A, B)** displayed a normal phenotype with a rigid and brownish cuticle while insects treated with ds*AgraLac2* exhibited abnormal phenotype with a soft cuticle lacking pigmentation (**C**). Representative pictures of insects were captured with an M205 FA fluorescence stereomicroscope (Leica) with FusionOptics^TM^. Scale bar = 2 mm.

The mock-treated larvae displayed normal morphology, whereas the ds*AgraLac2*-treated larvae exhibited a bloated body, presumably due to fat body accumulation accompanied by the increase of body volume without apparently cuticle growth (Figures 6A,B,A’,B’). Structures similar to the female ovipositor or male aedeagus were observed in both normal and abnormal larvae; however, in abnormal larvae the copulatory organs exhibited malformed phenotypes (Figures 6A”,B”). Notably, ds*AgraLac2*-treated adult insects exhibited an altered phenotype. The insects displayed physical deformities such as imperfectly formed hindwings and elytra, which were fenestrated and wrinkled. The elytra were also shorter, less pigmented and less rigid than those of the control insects. In addition, the ds*AgraLac2-*treated insects exhibited lack of sclerotization in the ventral abdomen and thorax as well as lighter pigmentation and softer cuticle in body regions such as legs and head compared to the control insects, which showed normal development and cuticle tanning process, resulting in a pigmented, rigid and sturdy body structure (Figure 7).

## Discussion

The exoskeleton (cuticle) plays several physiological and ecological roles and was an important acquisition that allowed the environmental expansion and evolutionary success of insects. It is required for muscle attachment and provides support to the internal organs and the skeletal elements necessary for locomotion. The exoskeleton also protects against injuries, predators and pathogens as well as avoids excessive water loss (Andersen, 2012; Zhu et al., 2016). Additionally, exoskeleton pigmentation is ecologically important for mating behavior, communication, mimicry, mimesis, and crypsis (Merrill et al., 2012, 2014; Olofsson et al., 2012; Shamim et al., 2014; Chouteau et al., 2016).

During insect growth and development, the cuticle formation involves several complex metabolic pathways in which Lac2 enzymes play critical roles. At each molt cycle, the old cuticle is replaced during the ecdysis and rapidly occurs the process of cuticle tanning leading to the darkening and hardening of the insect cuticle (Andersen, 2010, 2012; Moussian, 2010). Lac2 enzyme catalyzes the reactions of the cuticle tanning metabolic pathway, and its involvement in cuticle sclerotization and pigmentation has been demonstrated in several insect species (Arakane et al., 2005; Elias-Neto et al., 2010; Ye et al., 2015; Du et al., 2017). Moreover, maternal RNAi targeting *Lac2* has been shown to affect eggshell sclerotization and pigmentation of *Aedes albopictus*, resulting in deformed and fragile eggs, whereas maternal RNAi in *Plautia stali* leads to the production of eggs without any noticeable eggshell deformity but unable to hatch (Wu et al., 2013; Nishide et al., 2020). Accordingly, these studies indicate that in addition to cuticle tanning, *Lac2* is also involved in eggshell formation and egg hatchability in at least some insect species. Although the biological function of *Lac2* has been extensively studied in diverse insect species, its involvement in cuticle tanning, molting and development in *A. grandis* has not been explored. In the present study, we functionally characterized *AgraLac2* gene in an attempt to better understand its role in this important insect pest and to evaluate its potential as a target gene to be applied in RNAi approaches for controlling *A. grandis*.

We first investigated the expression pattern of *AgraLac2* throughout the life cycle of *A. grandis*. *AgraLac2* expression was detected in all developmental stages of *A. grandis* with increased expression levels in pupae and adults. This developmental expression profile is consistent with the function of *Lac2* in the insect cuticle tanning process, which is expected to be more pronounced particularly in the later stages of development. In agreement with our findings, transcripts of *Lac2* were expressed in all developmental stages of *A. gambiae* and *Aethina tumida* (Gorman et al., 2008; Powell et al., 2016). Similarly, the expression of *Lac2* was detected in the analyzed pupal and adult stages of *Tenebrio molitor* (Mun et al., 2020), *Monochamus alternatus* (Niu et al., 2008), *Apis mellifera* (Elias-Neto et al., 2010), *A. sinensis* (Du et al., 2017) and *T. castaneum* (Arakane et al., 2005). It is noteworthy that *Lac2* temporal expression is very dynamic and may be variable even within a given developmental stage. It has been proposed that *Lac2* expression is controlled by ecdysteroids, being induced concurrently with the increase in ecdysteroid titer that coincides with the pre-ecdysial cuticle tanning period (Elias-Neto et al., 2010, 2013; Du et al., 2017).

We further evaluated the localization of the AgraLac2 protein in third-instar larvae through immunohistochemical analyses. The epidermal cells found at the base of the procuticle are the principal cells responsible for the synthesis of Lac2 and as expected AgraLac2 was detected throughout the epidermal cells and cuticles of *A. grandis* larvae. This finding is in agreement with the observed *AgraLac2* transcripts expression, which was detected in third instar-larvae, implying the involvement of AgraLac2 in larval cuticle tanning even though larvae display much less tanned cuticle than pupae and adults. Interestingly, we observed that AgraLac2 was also expressed in the tracheal system, showing that AgraLac2 is present in internal cuticle structures of *A. grandis* larvae.

We demonstrated that larvae injected with ds*AgraLac2* exhibited serious phenotypical defects and abnormal development due to disruption of cuticle tanning process, leading to insect death during or after molting. Our results are consistent with previous reports demonstrating that *Lac2* is required for cuticle tanning in many insect species from different orders, including *Diabrotica virgifera virgifera* (Alves et al., 2010), *T. castaneum* (Arakane et al., 2005), *Cylas brunneus* (Christiaens et al., 2016), *A. sinensis* (Du et al., 2017), *A. mellifera* (Elias-Neto et al., 2010), *Riptortus pedestris*, *Nysius plebeius*, *Megacopta punctatissima* (Futahashi et al., 2011), *P. stali* (Nishide et al., 2020), *M. alternatus* (Niu et al., 2008), *Cylas puncticollis* (Prentice et al., 2015), *Nilaparvata lugens* (Ye et al., 2015), *A. tumida* (Powell et al., 2016), *Leguminivora glycinivorella* (Meng et al., 2018), *Chrysomela populi* and *Phaedon cochleariae* (Pentzold et al., 2018). Altogether, these studies indicate a conserved biological function of *Lac2*.

Since *Lac2* is implicated in an essential metabolic pathway, the depletion of *Lac2* transcripts is expected not only to compromise insect development but also to cause insect mortality as reported by several studies. Arakane et al. (2005) demonstrated that suppression of *Lac2* transcripts in *T. castaneum* affected cuticle tanning and resulted in insect mortality, suggesting that *Lac2* plays a key role in the sclerotization and pigmentation of larval, pupal and adult cuticles (Arakane et al., 2005). Likewise, knockdown of *Lac2* in third-instar nymphs of *P. stali* and *N. lugens* affected cuticle tanning, molting and resulted in 100% of insect lethality (Ye et al., 2015; Nishide et al., 2020). Furthermore, RNAi-mediated *Lac2* silencing in *A. sinensis* and *A. mellifera* impaired cuticle sclerotization and pigmentation leading to phenotypic abnormalities that affected adult eclosion rate (Elias-Neto et al., 2010; Du et al., 2017).

Currently, the control of *A. grandis* is essentially based on broad-spectrum chemical insecticides and no transgenic Bt cotton resistant to this insect pest is available commercially. Therefore, innovative and alternative management tools directed against *A. grandis* are urgently needed. The development of RNAi-based technologies for the management of insect pests provides new tools for crop protection that can be highly species-specific and environmentally friendly. Genes that are essential for insect survival, fecundity and/or development have been proposed as good RNAi targets to be applied in the control of agricultural pests and vectors of diseases (Bally et al., 2016; Coelho et al., 2016; Murphy et al., 2016; Macedo et al., 2017; Niu et al., 2017; Knorr et al., 2018; Lopez et al., 2019). We observed that disruption of cuticle tanning metabolic pathway by suppression of *AgraLac2* transcripts caused severe morphological deformations in *A. grandis* and negatively affected insect development and molting, inducing high mortality rates. Based on these findings we provide evidence that *AgraLac2* may be a suitable target gene for RNAi-mediated control of *A. grandis*.

After ingestion by the insect, the dsRNA is taken up by gut cells from the digestive tract and subsequently occurs the systemic spreading of the RNAi signal to other cells or tissues to induce the environmental RNAi response (Hanneke and Smagghe, 2010). Considering that the availability of adequate amounts of intact dsRNA for insect ingestion and the dsRNA protection against the action of intra- and extracellular dsRNases are important factors that may contribute to high RNAi efficiency, diverse methods for dsRNA delivery have been proposed to improve the RNAi response in the target insect pests and effectively to protect plants against their attack (Zotti et al., 2018). Further studies should be performed to explore the feasibility of RNAi pest control approaches, such as transgenic cotton plants expressing ds*AgraLac2* into the nuclear or the plastid genome as well as engineered fungi, bacteria, and virus expressing ds*AgraLac2* or ds*AgraLac2* complexed with nanoparticles as a dsRNA delivery method for foliar application on cotton leaves. Importantly, for field applications, the impact of RNAi-based pest control technologies to non-target organisms and environment needs to be cautiously assessed before they can be safely used on crops (Arpaia et al., 2020; Bachman et al., 2020; Mezzetti et al., 2020; Romeis and Widmer, 2020). Consequently, future approaches relying on *AgraLac2* dsRNA to control *A. grandis* will require a comprehensive risk assessment to ensure their biosafety.

Some melanin biosynthesis pathway genes, including *tyrosine hydroxylase* (*TH*), *phenylalanine hydroxylase* (*PAH*) and *Lac2* have been associated with the melanotic encapsulation immune responses triggered by microorganism infections in certain insect species (Infanger et al., 2004; Qiao et al., 2016; Du et al., 2017; Chen et al., 2018). A recent study demonstrated that *Lac2* silencing in *A. sinensis* significantly reduced the resistance to the exogenous bacteria *Serratia marcescens* and *Bacillus bombyseptie*, implying that *Lac2* participates in melanotic immune responses (Du et al., 2017). Another study showed that *Lac2* is crucial for the antifungal host defense of *T. castaneum* adults. Knockdown of *Lac2* in adults of *T. castaneum* impaired the host defense against the entomopathogenic fungi, *Beauveria bassiana* and *Metarhizium anisopliae*, drastically decreasing the survival of the insects upon fungi infection (Hayakawa et al., 2018). We speculate that *AgraLac2* also could be involved in this mechanism of protection against exogenous pathogen infections in *A. grandis*. In addition to the morphological defects and mortality of the insects due to *AgraLac2* silencing, the insects could become more susceptible to microorganism infections using RNAi-based strategies with *AgraLac2* as a target gene. Therefore, such strategies could be used in association with biological control agents to achieve more effective management of *A. grandis* populations. Nonetheless, whether *AgraLac2* is implicated in immune responses against microorganisms still needs to be investigated.

In summary, our study provides the first insights into the physiological and developmental functions of *AgraLac2* in *A. grandis*. Our data demonstrated that *AgraLac2* silencing affected cuticle tanning and development of insects, resulting in lethal phenotypes. Thus, *AgraLac2* may be a potential target for the development of innovative strategies for *A. grandis* control.

## Data Availability Statement

The raw data supporting the conclusions of this article will be made available by the authors, without undue reservation.

## Author Contributions

AF and MG-S conceived the original research plan. DM-S cloned the gene *AgraLac2*. AF performed the identification and sequence analysis of *AgraLac2*, the dsRNA synthesis and the RT-qPCR analyses. FA performed the phylogenetic three. AF, LM, FF, and RC performed the RNAi experiments under the supervision of WT. JA performed the statistical analyses. CM-P and JE performed the fluorescent immunolocalization assays. MS performed the immunostaining assays. DP, AF, and JA analyzed the results. DP wrote the manuscript. AF, JA, and MG-S reviewed the manuscript. IL-T, MS, and MG-S supervised the work. All authors contributed to the article and approved the submitted version.

## Conflict of Interest

The authors declare that the research was conducted in the absence of any commercial or financial relationships that could be construed as a potential conflict of interest.

## Footnotes

1 https://www.ncbi.nlm.nih.gov/

2 https://mafft.cbrc.jp/alignment/server/

3 https://itol.embl.de/

4 https://pfam.xfam.org/

5 http://meme-suite.org/tools/meme

6 http://weblogo.berkeley.edu/logo.cgi

7 http://rnaidesigner.invitrogen.com/rnaiexpress

8 http://miner.ewindup.info
