# Supplementary material for: RNAi-Mediated Suppression of Laccase2 Impairs Cuticle Tanning and Molting in the Cotton Boll Weevil (Anthonomus grandis)
Source: Front Physiol. 2020 Nov 16;11:591569. doi: 10.3389/fphys.2020.591569 (PMC7717984; doi:10.3389/fphys.2020.591569)
Supplement: Supplementary file 1 [file Data_Sheet_1.PDF]

Supplementary Information

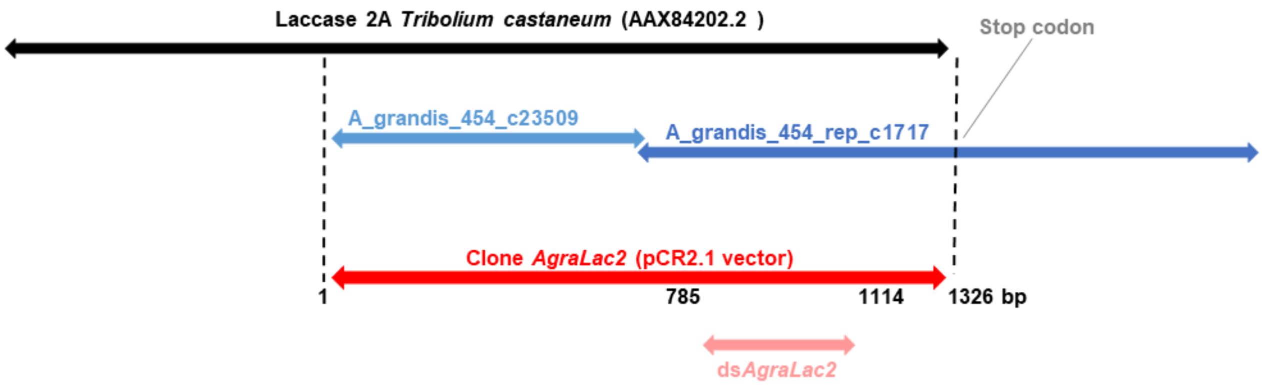

Figure S1. Schematic representation of the cloning strategy used to obtain a partial sequence of *Lac2* from *A. grandis* (*AgraLac2*).

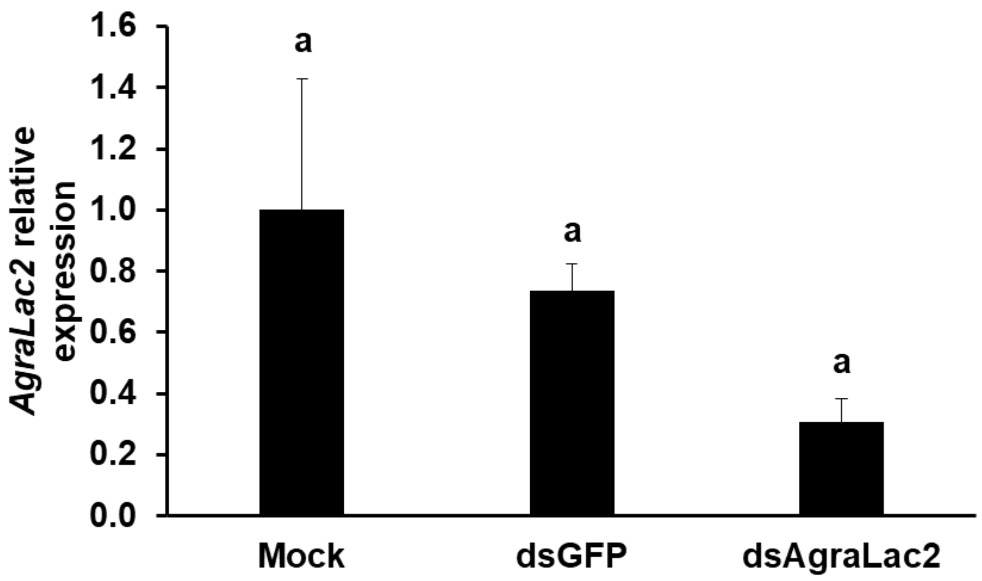

Figure S2. Relative transcript levels of *AgraLac2* after dsRNA exposure. Relative transcript levels of *AgraLac2* gene in *A. grandis* larvae at 2 days after water (mock), ds*GFP* or ds*AgraLac2* injection evaluated by RT-qPCR. The expression data were normalized using *gapdh* and *β-tubulin* as reference genes. Values shown are the means and standard errors (±SE) of two biological replicates each with three technical replicates. Data were analyzed by one-way ANOVA followed by a post hoc multiple comparisons test (Tukey's HSD test). Treatment groups with different letters are significantly different ($P < 0.05$).

Supplementary Data S2: Contig sequences of *Lac2* retrieved from *A. grandis* transcriptome

>A_grandis_454_c23509

AGATTTGACTACTCATGTTATTTTGCTTTCTGATTGGATGCACGAAGATGCTGCCGAGAGATTCCCTGGT
AGATTGGCTGTTAACACCGGTCAAGATCCTGAAAATTTACTCATCAACGGAAAAGGTCAATTCCGTGATC
CCAATACCGGTTTCATGACCAACACCCCCTTGGAAATTTTCACGATGACTCCCGGAAAACGGTACAGATT
CCGTATGATCAACTCCTTGGCGTCAGTCTGTCCTGCTCAWTTAACAGTCCAGGGACACACGTTGACTCTT
ATTGCCACCGATGGTGAACCTGTCCATCCAGTTAACGTGAACACCGTAATTTCTTTCTCAGGAGAAAGAT
ACGATTTTGTAATCAACGCGGACCAATCAGTAGGAGCCTACTGGATCCAAGTGAGAGGCTTAGGAGAATG
TGGTATCAGAAGGGTACAGCAATTGGCTATTTTTGAGATACTACAAGGGACCATATACTCCATTCACCCA
AGCACCTACTTATGACTTCGGTATTCCTACAGGAGTGGTTCTGAATCCCCTAGACGCTATTTGTAATAAA
CCCAGGAAAGACGCAGTGTGCGTCNGTCAGCTAACGAATGCCAAAAAGTGGACGAAGCCATTTGCAAGAA
CGGCCTGATG

>A_grandis_454_rep_c1717

ATATTTTGAGATACTACAAGGGACCATATACTCCATTCACCCAAGCACCTACTTATGACTTCGGTATTCC
ACAGGGAGTGGWACTTAATCCTTTGGACGCAATTTGTAATGAAACCAGAGCAGATGCCGTTTGTATCAGT
CAATTGAAAAATGCCAGAGAAGTGGACAGGGCGCTGCTGATTGAAAAACCAAATGTGAAGATATTTTTGC
CATTCAGATTCCACGTTTATACACCGGAGGATCTTTTTAATCCTAACAGTTACAACAGACATTTAGTTGC
GCCAAACGGAGACCACGTAATCAGTTTAATCGACGAAATCTCGTACATGGCTGCGCCAGCTCCGCTTCTA
TCTCAGTACGATGAAATTGACCCCGAGCAATTCTGTAATGGAGATAACAGACCACCCAATTGTGGACCTA
ACTGCATGTGTACCCACAAAATTGATATTCCATTAAATGCGATTGTCGAAGTTGTTCTGGTTGATGAAGT
TCAACAACCGAACTTATCCCATCCGTTCCATTTGCACGGTTATGCTTTTAATGTGGTCGGTATTGGTCGC
TCACCTGACACAAGTGTCAAGAAAATTAACTTAAAACACGCTTTGGATCTGGATCGGAGAGGTCTGTTGC
ATAGGCACTTTGATCTGCCACCCGGTAAAGACACCATTGCTGTACCAAACAATGGATACGTAATCTTCAG
ATTCCGAGCAGACAATCCTGGTTTCTGGCTTTTCCACTGTCACTTCTTGTTCCACATAGTTATAGGAATG
AATTTGGTCCTGCAAGTGGGTACACATGCAGACATACCACCAGTGCCACCGAATTTCCCTACTTGCGGCG
ATCACACGCCACGAATCAATTTAGATCCAACGAAAATATGAAGACTTAATACCTAGTATTTTTTTTAAGA
TTAAGGGTCCTTTTTAGTATAGGTTGGTGTAAATAAGAGTCAAGTCAAAGGTTTGGTAAGTATTACGAGT
TAATATATTTTGTGAAAAAATGTACAGGGTTATGCTACTCATGTATATTATTTCAGGTAGGTATAATAAC
GCAATGTATATAATTTTTTACTAGCACAATATTGTAAATATAACATATTTACACGATCTTATTTGTCACC
AACTGCTAGTATATTGTAGAGTTACCAAATTAATATTCTGAATATTTGGATTAAGTCTTGCCATCCAAAA
ATGTCGAAAAATCAGTATTCTAAATTGAGTGTTTTCCTATATTATGTATTTTATTTATCTGATATTTATC
TGTACTGTACCAAATGGTCTTTATCGCCATATTATTATATATAATTTTAGATATTCTACCTCTTTCTATG
TGTTTWTTTTTTGTTTTTTTTAACTATCACTATTGGTGCTTTCTAAAACTTATAAATCAATTATTTGAGT
TAAAATCTTTAAATATTTTTTGATACTTTTCATAATAAGAAAAATGTTTTGGTTAATATTTTTAATATGG
GAAATATTTAAAGATTTAAAGGTATGAACCCTTTCGTAAATAAATTATTTCTTCCAGGAAGTAAGCTAAG
GTCTTTAATGTAAAAATTCTTATAGTTATAGTAGAAATTTTATGATTATGTCATTGGAAATGTTACCAAC
ATAACGAAGTAAATAAATATGAATTTTATCACATCTATACCACAAAAATAGGCACTTCATAGTACATTTT
TTGGTAAAAGAACCACCTAGTCTATTACAAAACCAAGACGAATATGAAAATAAGTCATCGTTGTAGATTT
GTATATAAAATAATAGTGTAAAGTACCAATGTATTTAAAGTAATAAAATTTGTGACTATATTTATGT

TGA - Stop codon

Supplementary Data S3: *AgraLac2* partial mRNA sequence obtained from the consensus of contig sequences (A_grandis_454_c23509 and A_grandis_454_rep_c1717) and the cloned *AgraLac2* sequence.

>*AgraLac2* partial, mRNA

```
GATTTGACTACTCATGTTATTTTGCTTTCTGATTGGATGCACGAAGATGCTGCCGAGAGATTCCCTGGTA
GATTGGCTGTTAACACCGGTCAAGATCCTGAAAATTTACTCATCAACGGAAAAGGTCAATTCCGTGATCC
CAATACCGGTTTCATGACCAACACCCCCTTGGAAATTTTCACGATGACTCCCGGAAAACGGTACAGATTC
CGTATGATCAACTCCTTGGCGTCAGTCTGTCCTGCTCAATTAACAGTCCAGGGACACACGTTGACTCTTA
TTGCCACCGATGGTGAACCTGTCCATCCAGTTAACGTGAACACCGTAATTTCTTTCTCAGGAGAAAGATA
CGATTTTGTAATCAACGCGGACCAATCAGTAGGAGCCTACTGGATCCAAGTGAGAGGCTTAGGAGAATGT
GGTATCAGAAGGGTACAGCAATTGGCTATTTTGAGATACTACAAGGGACCATATACTCCATTCACCCAAG
CACCTACTTATGACTTCGGTATTCCACAGGGAGTGGTACTTAATCCTTTGGACGCAATTTGTAATGAAAC
CAGAGCAGATGCCGTTTGTATCAGTCAATTGAAAAATGCCAGAGAAGTGGACAGGGCGCTGCTGATTGAA
AAACCAAATGTGAAGATATTTTTGCCATTCAGATTCCACGTTTATACACCGGAGGATCTTTTTAATCCTA
ACAGTTACAACAGACATTTAGTTGCGCCAAACGGAGACCACGTAATCAGTTTAATCGACGAAATCTCGTA
CATGGCTGCGCCAGCTCCGCTTCTATCTCAGTACGATGAAATTGACCCCGAGCAATTCTGTAATGGAGAT
AACAGACCACCCAATTGTGGACCTAACTGCATGTGTACCCACAAAATTGATATTCCATTAAATGCGATTG
TCGAAGTTGTTCTGGTTGATGAAGTTCAACAACCGAACTTATCCCATCCGTTCCATTTGCACGGTTATGC
TTTTAATGTGGTCGGTATTGGTCGCTCACCTGACACAAGTGTCAAGAAAATTAACTTAAAACACGCTTTG
GATCTGGATCGGAGAGGTCTGTTGCATAGGCACTTTGATCTGCCACCCGGTAAAGACACCATTGCTGTAC
CAAACAATGGATACGTAATCTTCAGATTCCGAGCAGACAATCCTGGTTTCTGGCTTTTCCACTGTCACTT
CTTGTTCCACATAGTTATAGGAATGAATTTGGTCCTGCAAGTGGGTACACATGCAGACATACCACCAGTG
CCACCGAATTTCCCTACTTGCGGCGATCACACGCCACGAATCAATTTAGATCCAACGAAAATATGA
```

Supplementary Data S4. Predicted protein AgraLac2

>Predicted protein AgraLac2, partial

```
DLTTHVILLSDWMHEDAAERFPGRLAVNTGQDPENLLINGKGQFRDPNTGFMTNTPLEIFTMTPGKRYRF
RMINSLASVCPAQLTVQGHTLTLIATDGEPVHPVNVNTVISFSGERYDFVINADQSVGAYWIQVRGLGEC
GIRRVQQLAILRYYKGPYTPFTQAPTYDFGIPQGVVLNPLDAICNETRADAVCISQLKNAREVDRALLIE
KPNVKIFLPFRFHVYTPEDLFNPNSYNRHLVAPNGDHVISLIDEISYMAAPAPLLSQYDEIDPEQFCNGD
NRPPNCGPNCMCTHKIDIPLNAIVEVVLVDEVQQPNLSHPFHLHGYAFNVVGIGRSPDTSVKKINLKHAL
DLDRRGLLHRHFDLPPGKDTIAVPNNGYVIFRFRADNPGFWLFHCHFLFHIVIGMNLVLQVGTHADIPPV
PPNFPTCGDHTPRINLDPTKI
```

Table S1. Species distribution of BLASTx matches of *A. grandis* contigs.

***A. grandis* contig identification**	**BLASTx matches**	**Acession number**	**Identity (%)**	**E value**	**Query Cover (%)**
A_grandis_454_c23509	laccase 2 [*Rhynchophorus ferrugineus*]	QGK89547.1	89.10	1.00E-98	96
	laccase2 [*Drosophila busckii*]	ALC45418.1	78.00	8.00E-93	77
	laccase 2 [*Phaedon cochleariae*]	AWK23446.1	85.90	4.00E-92	96
	multicopper oxidase 2 [*Nilaparvata lugens*]	AKN21380.1	82.69	5.00E-91	94
	laccase 2 isoform X2 [*Contarinia nasturtii*]	XP_031632951.1	83.33	6.00E-91	94
	laccase 2 precursor [*Tribolium castaneum*]	NP_001034487.2	83.23	6.00E-91	93
	laccase 2 [*Monochamus alternatus*]	ABU68466.1	83.23	7.00E-91	94
	laccase 2 isoform X3 [*Contarinia nasturtii*]	XP_031632952.1	83.33	7.00E-91	94
A_grandis_454_rep_c1717	laccase 2 [*Rhynchophorus ferrugineus*]	QGK89547.1	88.36	6.00E-171	48
	laccase 2 [*Phaedon cochleariae*]	AWK23446.1	84.38	6.00E-162	47
	laccase 2 [*Chrysomela populi*]	AWK23445.1	83.33	2.00E-160	47
	laccase 2 precursor [*Tribolium castaneum*]	NP_001034487.2	81.85	2.00E-156	48
	laccase 2 isoform A [*Anopheles gambiae*]	AAX49501.1	82.17	1.00E-152	47
	laccase 2 isoform A [*Anopheles sinensis*]	KFB50921.1	82.17	2.00E-152	47
	laccase 2 [*Anopheles sinensis*]	ARG47519.1	81.82	2.00E-151	47
	laccase 2 [*Drosophila mojavensis*]	XP_002005808.1	82.11	8.00E-151	47
